# Nutritional regulation of muscle protein synthesis with resistance exercise: strategies to enhance anabolism

**DOI:** 10.1186/1743-7075-9-40

**Published:** 2012-05-17

**Authors:** Tyler A Churchward-Venne, Nicholas A Burd, Stuart M Phillips

**Affiliations:** 1 Department of Kinesiology, McMaster University, Hamilton, ON, Canada

**Keywords:** Nutrition, Muscle, Anabolic intramuscular signaling, Hypertrophy

## Abstract

Provision of dietary amino acids increases skeletal muscle protein synthesis (MPS), an effect that is enhanced by prior resistance exercise. As a fundamentally necessary process in the enhancement of muscle mass, strategies to enhance rates of MPS would be beneficial in the development of interventions aimed at increasing skeletal muscle mass particularly when combined with chronic resistance exercise. The purpose of this review article is to provide an update on current findings regarding the nutritional regulation of MPS and highlight nutrition based strategies that may serve to maximize skeletal muscle protein anabolism with resistance exercise. Such factors include timing of protein intake, dietary protein type, the role of leucine as a key anabolic amino acid, and the impact of other macronutrients (i.e. carbohydrate) on the regulation of MPS after resistance exercise. We contend that nutritional strategies that serve to maximally stimulate MPS may be useful in the development of nutrition and exercise based interventions aimed at enhancing skeletal muscle mass which may be of interest to elderly populations and to athletes.

## **Introduction**

The synergistic effects of amino acid provision and resistance exercise on skeletal muscle protein synthesis rates (MPS) are now well described (for reviews see: [1,2]). Consuming dietary amino acids after resistance exercise stimulates an increase in MPS and is necessary to shift net protein balance (defined as MPS minus muscle protein breakdown (MPB)) from negative (net protein loss) to positive (net protein gain) [3]. In healthy individuals, feeding-induced changes in MPS are ~3–5 times greater over the course of any given day than measurable changes in MPB, demonstrating that MPS is highly responsive, regulated, and represents the primary driver of changes in muscle net protein balance. As such, it would follow that for chronic elevations in net muscle protein balance to result in gains in muscle mass, changes in MPS are highly relevant. We do not contend that MPB is a trivial biological process; MPB assists in maintenance of intracellular amino acid levels, and likely plays a role in maintaining muscle protein quality by removing damaged proteins and allowing their constituent amino acids to be used for the synthesis of new functional muscle proteins. Consequently, we propose that nutritional interventions that enhance MPS may be of great scientific and clinical interest as a strategy to promote positive muscle protein balance and eventual muscle protein accrual. Further, these interventions may be of interest to athletes concerned with enhancing the adaptive response of skeletal muscle to chronic exercise training. Current research has demonstrated that factors such as the dose of dietary protein/essential amino acids (EAA) ingested [4,5], protein food source (i.e. whey, soy, micellar casein) [6-9], and timing of protein/EAA intake [9-11] impact the magnitude (and possibly the duration) of MPS in response to feeding and resistance exercise. Other research has focused on the ability to enhance MPS by providing increased amounts of leucine [12-14] or arginine [15] within an amino acid containing solution. Lastly, the influence of consuming mixed macronutrients on muscle protein metabolism [16-20] has also received some attention. The purpose of this review is to discuss the nutritional regulation of human MPS and provide an update on nutritional strategies that may serve to maximize MPS with feeding and resistance exercise.

### **Redefining the ‘window of anabolic potential’ after resistance exercise**

**Figure 1:**
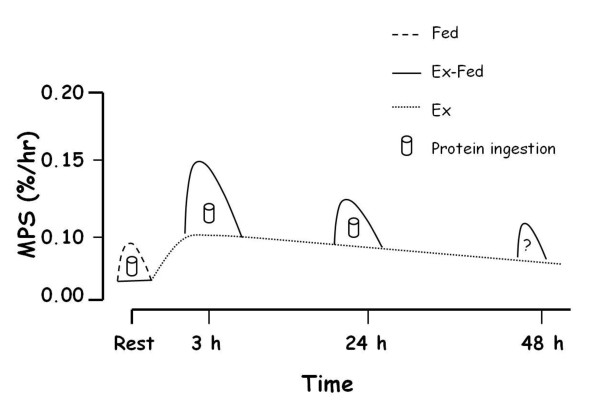
**Resistance exercise stimulates a prolonged elevation of muscle protein synthesis (MPS) that can remain elevated for ≥ 24 h (dashed lines).** Thus, we propose that protein ingestion at any point during this enhanced period of ‘anabolic potential’ will be additive to these already elevated exercise mediated rates (solid line).

**Figure 2:**
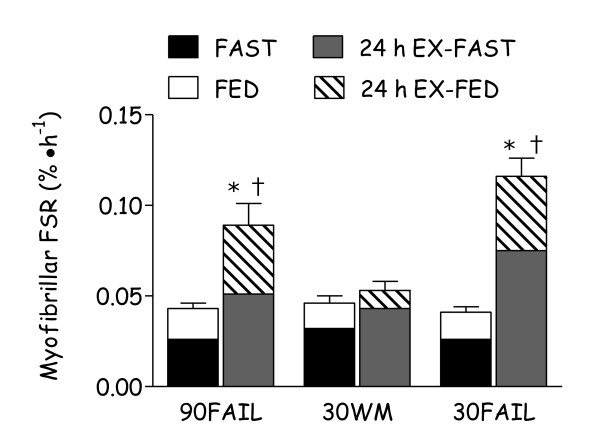
**Enhanced amino acid sensitivity of myofibrillar protein synthesis (FSR) persists for up to 24 h only after resistance exercise that results in maximal muscle fibre activation induced by high load low volume resistance exercise (90FAIL) or low load high volume resistance exercise (30FAIL).** 30WM represents a worked-match control to the 90FAIL condition that did not result in full muscle fibre recruitment. The change in myofibrillar protein synthesis rates are determined from the transition from fasting (FAST) to feeding 15 g of protein at rest (FED) or 24–27 h after resistance exercise in the fasting- (24 h EX FAST) or fed-state (24 h EX-FED). *Significantly different from FED (P < 0.05). †Significantly different from 30WM (P < 0.05). Adptated from Burd and colleagues [27].

Although the amino acid mediated increase in MPS is transient, lasting only a few hours at most [21-23], the contractile activity associated with intense resistance exercise results in increased rates of MPS that are sustained for ~48 h in the fasted state in young participants [24]. It is now unequivocal that immediate post-exercise amino acid provision is an effective nutrition based strategy to enhance MPS above rates observed with exercise alone [3,5,25]. The importance of early post-exercise protein ingestion relates to the fact that exercise-mediated increases in rates of MPS are greatest immediately after exercise (~100 – 150% above basal rates) [2], and thus the synergistic effects of exercise and feeding on MPS are likely greatest during this time-period. However, since resistance exercise increases MPS for up to ~48 h [24] consumption of dietary amino acids 24 - 48 h post-exercise recovery would also likely convey the same synergistic effects on MPS as those that are observed when amino acids are provided immediately after resistance exercise [7,8,25,26]. As shown in Figure 1, the synergistic enhancement of pre-existing resistance exercise-induced elevations in MPS by protein provision is greatest immediately post-exercise and wanes over time, but may still be present up to 48 h later. We have recently shown that feeding 15 g of whey protein, a less than optimally effective dose of protein for maximizing MPS [5], ~24 h after acute resistance exercise results in a greater stimulation of myofibrillar (contractile proteins of skeletal muscle) protein synthesis than the same dose provided at rest [27] (Figure 2). However, the effect of enhanced sensitivity to protein ingestion induced by prior resistance exercise performed 24 h earlier was independent of the amount of weight lifted. Specifically, resistance exercise was performed at a relatively high load (90FAIL) or low load (30FAIL), but both regimens were performed to volitional fatigue. Thus, irrespective of exercise load, the ultimate result was eventual similar increases in muscle fibre recruitment [28]. Future research should examine if there are age-related differences in the ability of resistance exercise to convey an enhanced sensitivity of MPS to protein ingestion when consumed ~24 h after exercise, and whether this effect is influenced by the type of protein consumed as these results would be relevant to increasing our understanding of the factors involved in age-related muscle loss.

Overnight nutrient provision may also represent an effective nutritional strategy to stimulate MPS, and thus increase the ‘window of anabolic opportunity’ by promoting a greater muscle net protein balance over the course of 24 h. Previous research has shown that overnight MPS rates are quite low [29], however both intragastric protein provision during sleep [30], and oral protein ingestion after resistance exercise immediately before bed [31] are followed by normal protein digestion and absorption kinetics and an overnight stimulation of MPS. Therefore, we contend that properly planned protein/EAA provision, not only immediately after, but up to ~24 h following exercise should be carefully considered as a dietary strategy to maximally stimulate exercise-induced rates of MPS.

### **Mechanisms underpinning the enhanced sensitivity of skeletal muscle after exercise**

Dietary amino acids and insulin are major nutrient-regulated effectors of MPS and MPB and recent work has shed light on the molecular pathways involved in regulating the amino acid and contraction-induced increase in MPS. A comprehensive review of the molecular regulation of MPS in response to nutrition and exercise is beyond the scope of this article but can be found elsewhere [32]. The protein kinase mTORC1 serves as a critical point of integration from a wide range of signals that promote MPS, including dietary amino acids [33] and muscle contraction [34]. Specifically, mTORC1 regulates MPS by phosphorylation of downstream protein effectors such as p70S6k and 4E-BP1 that are involved in translation initiation of MPS. Further, blocking mTOR activity with the drug rapamycin blocks both the contraction [34], and EAA [33] mediated increase in human MPS, demonstrating the essentiality of this kinase in the regulation of MPS. To date, several studies have demonstrated that amino acid provision after resistance exercise and the subsequent increase in MPS are associated with enhanced phosphorylation of components of the mTOR signaling cascade above levels that are observed following exercise without nutrients [26,35-37]. However, dissociation between direct measures of rates of MPS and the extent of muscle anabolic signaling molecule phosphorylation has been reported previously [13,38]. Further, exactly how amino acids are able to activate mTORC1 to increase MPS is not understood, although MAP4K3 [39], vacuolar sorting protein 34 (Vps34) [40,41], and Rag GTPases [42] are signaling proteins that are candidate amino acid ‘sensors’ capable of responding to changes in amino acid concentration with subsequent activation of mTORC1. In addition, the mRNA expression of select skeletal muscle amino acid transporters such as LAT1 (SLC7A5), SNAT2 (SLC38A2), CD98 (SLC3A2), and PAT1 (SLC36A1) has been reported to be increased following EAA ingestion [43] and resistance exercise [44] in human skeletal muscle. These transporters may play an important role in the regulation of human muscle protein metabolism based on their ability to transport amino acids across the cell membrane, and relay signals to downstream targets thought to regulate MPS [45]. An increase in the protein levels of some of these amino acid transporters has also been observed following EAA ingestion [43] and resistance exercise [44], however it is currently unclear whether increases in mRNA and protein expression of these transporters are associated with enhanced amino acid transport capacity. Clearly, further research is needed to define the functional and physiological significance of these transporters in the nutrition and exercise mediated regulation of MPS.

### **Optimizing MPS: the role of protein source**

The ingestion of dietary proteins including whey [5-8,21,27,46,47], egg albumin [5], soy [7,8], casein [6,8], and beef [48,49] are all able to stimulate MPS. However, dietary proteins from different sources differ in their capacity to stimulate MPS both at rest [6-8] and following resistance exercise [7,8]. For example, work from our lab has shown that whey protein [8] and bovine milk [7] promote greater increases in MPS after acute resistance exercise than does consumption of an equivalent amount of plant-based soy protein despite the fact that these protein sources have protein digestibility-corrected amino acid scores (PDCAAS) above 1.0. The limitations of the PDCAAS scoring system and the artificial truncation at 1.0, when some proteins have a PDCAAS of > 1.0, has been discussed in our previous review [50]. The mechanisms responsible for these differences are not entirely clear but may relate to important differences in the amino acid profile and/or amino acid availability due to differences in the digestion/absorption kinetics of the proteins. Whey protein is acid soluble and is associated with a very rapid, large, but transient increase in postprandial amino acid availability [6,51], while casein coagulates and precipitates when exposed to stomach acid and the resultant dairy curd is slowly released from the stomach resulting in a much more moderate but sustained rise in plasma amino acids [6,51]. Our lab has recently compared the effects of whey protein isolate to micellar casein on rates of MPS in elderly men [52]. Consistent with our previous findings in young subjects [8] we reported greater increases in blood leucine concentration and increases in both rested and post-exercise MPS after ingestion of 20 g of whey protein isolate than ingestion of micellar casein. This data corroborates our previous work showing that a rapid rate of amino acid appearance in the blood after feeding enhances MPS and anabolic cell-signaling after resistance exercise more than a slow rate of amino acid appearance [53], supporting the notion that protein digestion and absorption rate represents an important factor in the nutritional regulation of MPS in humans [8,47,51,52,54,55]. Our data on MPS are dissimilar, in some regards, to results obtained from studies of whole-body protein turnover [51,54,55], likely because skeletal muscle accounts for only ~30% of whole body protein synthesis [56] and turns over at a rate that is significantly less (~20-fold) than both splanchnic [57] and plasma proteins [58]. Interestingly, recent research suggests that the form of food (i.e. liquid vs. solid) may be an important factor regulating postprandial plasma amino acid availability [59]. For example, Conley and colleagues [59] showed greater increases in plasma amino acids that were more sustained following beverage administration as compared to the same supplement (i.e. energy and macronutrient matched) provided in solid food-form. These findings are interesting in light of the fact that the postprandial rise in plasma EAA [60] and/or leucine [61,62] appears to be key regulator of the postprandial rise in MPS, but more research is needed to determine the physiological relevance of food form as it pertains to the regulation of MPS.

### **Leucine as a nutrient signal in skeletal muscle**

Of the amino acids, the EAA are primarily responsible for stimulating MPS [63,64], whereas non-essential amino acids appear ineffective in this regard [65]. The branched-chain amino acid (BCAA) leucine appears unique among the EAA as a key regulator of translation initiation of MPS [66,67]. For example, leucine, but not isoleucine or valine can stimulate an increase in MPS through activation of the mTOR-p70S6k pathway in animals [66,68]. Work in cell culture utilizing C2C12 cells has demonstrated that leucine is the most potent among the EAA in its ability to increase the phosphorylation status of p70S6k, and the only EAA capable of increasing the phosphorylation status of mTOR and 4E-BP1 [69]. Taking these data into account, recent research has focused on utilizing leucine as part of a nutritional intervention to modulate MPS and/or muscle mass in humans. Tipton and colleagues [12] examined the effect of free leucine (3.4 g) added to whey protein (16.6 g) on rates of MPS after acute resistance exercise and reported no further increase in MPS with the addition of free leucine compared to that previously reported for 20 g whey protein. However, these data are not surprising in light of work from our group [5] and others [4] in which the dose–response relationship between protein/EAA ingestion and MPS was examined. Moore and colleagues [5] reported that MPS was maximally stimulated in young men with 20 g of high-quality protein after resistance exercise, with 40 g of protein resulting not in increased MPS above that observed with 20 g, but simply elevated levels of amino acid oxidation. Thus, ingestion of leucine in amounts greater than that found in a saturating dose (20–25 g whey protein containing 2.5-3.0 g leucine) of high quality protein, is unlikely to further stimulate an increase in the magnitude or duration of MPS. However, these data are taken from young healthy men weighing ~86 kg and the maximally effective dose of protein may be quite different in, for example, a ~50 kg female gymnast or a 120 kg bodybuilder. The elderly also represent a population that may require greater amounts of dietary protein and/or leucine to mount a robust increase in MPS in response to feeding [70,71]. Future research is needed to define the amount of leucine required to stimulate MPS in both young and elderly adults and to clearly establish the role of other EAA in the regulation of MPS with feeding and resistance exercise.

### **Post-exercise nutrition for the elderly**

Defining nutritional interventions that maximally stimulate rates of MPS are of interest in the development of therapeutic strategies designed combat age-related muscle loss (sarcopenia). The cause of sarcopenia is likely multi-facted [72], however some evidence suggests that that the elderly are ‘resistant’ to the anabolic effects of amino acids [4,73] and resistance exercise [74], and to the anti-proteolytic effects of insulin [75]. For example, Kumar and colleagues [74] reported an age-related blunting of the MPS response in the post-absorptive state following acute resistance exercise performed over a range of intensities (20-90% 1RM) when measured over 1-2 h post-exercise recovery. However, since free-living individuals typically eat after resistance exercise, it can only be speculated whether the same blunted MPS response between young and old would have been observed in the fed-state.

Despite the diminished response to amino acid provision and exercise in the elderly, it appears that the additive effects of feeding and resistance exercise on rates of MPS are preserved in this population, with several studies showing that combined feeding and exercise results in greater increases in MPS than feeding alone [48,52,76]. Our lab has recently examined the dose–response relationship between whey protein ingestion and myofibrillar protein synthesis under both rested and post-resistance exercise conditions in the elderly [76]. Contrary to young participants in whom MPS is maximally stimulated after resistance exercise with ~20 g of protein, 40 g of protein increased rates of MPS in the elderly more than 20 g when consumed after resistance exercise [76], suggesting that the elderly may benefit from a greater amount of amino acids and/or leucine after resistance exercise to maximize myofibrillar protein synthesis. In support of the elderly responding to greater amounts of leucine, Katsanos and colleagues (2006) reported that a 6.7 g mixture of EAA containing 26% leucine was unable to promote an increase MPS above basal levels in the elderly; however, when the leucine content of the same EAA mixture was increased to 41%, MPS was stimulated above basal to the same extent as that observed in young subjects [70]. These findings suggest that amino acid composition, and not simply total EAA is of key importance in determining the postprandial response of MPS in elderly muscle. However, the efficacy of free leucine supplementation with meal feeding as a strategy to augment muscle mass in the elderly is not currently supported. Verhoeven and colleagues (2009) examined the efficacy of long-term leucine supplementation on skeletal muscle mass in elderly subjects and reported that supplemental leucine (7.5 g per day with meals) for a 12-week period did not increase skeletal muscle mass or strength when compared to an energy-matched placebo [77]. However, this was only in subjects who were consuming standard meals and meal-induced gains in lean mass from feeding alone, without resistance exercise are likely to be small, especially over a period of 12 weeks. Further, the leucine supplementation was associated with declines in circulating valine and isoleucine which could have become limiting for the stimulation of MPS [77]. Studies in animals have shown that leucine provision leads to a decline in circulating EAA and reduces the duration of the amino acid mediated increase in MPS [78]; however, when this decrease is prevented and basal amino acid concentrations are maintained, the response of MPS to amino acid provision lasts ~2 hours [79]. Overall, additional dietary leucine, which may be obtained from high quality proteins and not necessarily in crystalline form, may be of some benefit to the elderly from the perspective of increasing MPS [70,71]. More research is needed to examine the effect of leucine-enriched amino acid provision in the early time period after resistance exercise on MPS and gains in lean mass following more long-term training.

Recently, Smith and colleagues (2011a, 2011b) have examined the role of supplemental (4 g day for 8 weeks) omega 3 polyunsaturated fatty acids on rates of MPS and the activation of signaling proteins within the mTOR-p70S6k pathway in both young and middle aged [80], and elderly subjects [81]. In all age groups examined, supplementation with omega 3 fatty acids significantly increased the magnitude of the amino acid/insulin induced stimulation of MPS and phosphorylation of mTOR [80,81]. Although the mechanisms are currently unknown, these results suggest that omega 3 polyunsaturated fatty acids possess anabolic properties via their ability to enhance the sensitivity of skeletal muscle to amino acids and insulin, even in young healthy individuals [80,81]. Recently it has been shown that supplemental fish oil (2 g/day) is also able to enhance the adaptive response to chronic resistance exercise training by promoting increases in muscle strength in elderly women [82]. Future research should examine the role of supplemental omega 3 polyunsaturated fatty acids on lean muscle mass accrual following a period of chronic resistance exercise training in both the young and elderly.

### **Role of carbohydrate and insulin in the regulation of muscle protein metabolism**

Consumption of a typical mixed meal is generally associated with the ingestion of not only dietary proteins and amino acids, but also carbohydrates and lipids. While almost nothing is known about the impact of lipid-protein co-ingestion on direct measures of MPS with feeding and resistance exercise, Elliot and colleagues [83] reported that threonine and phenylalanine uptake (indicative of an anabolic response) was greater after ingestion of whole milk (8.2 g fat, 8.0 g protein, 11.4 carbohydrate: total 627 kcal) as compared to fat free milk or isocaloric control conditions that were devoid of fat. The reason for the greater anabolism after whole milk ingestion is not entirely clear; however, it may relate to the greater muscle perfusion, at least in that study. Previous studies have investigated the role of carbohydrate (CHO) in the regulation of human muscle protein metabolism [16-19,84]. Intake of CHO is associated with increased levels of circulating insulin, which has a strong inhibitory effect on MPB [38,85,86], and thus is able to improve net protein balance [16-19,84]. However, in the absence of amino acid intake, CHO intake does not result in a positive net protein balance [19,84]. Our lab has recently examined the effect of carbohydrate-protein co-ingestion as compared to protein intake alone on rates of MPS and MPB after acute resistance exercise in young men [17]. Subjects consumed 25 g of whey protein or 25 g of whey protein with 50 g of added CHO as maltodextrin. Area under the plasma insulin curve was ~5-fold higher following protein-carbohydrate co-ingestion, however measures of limb blood flow, MPS, and MPB at rest and after resistance exercise were not different as compared to protein alone [17]. Therefore, when protein intake is of sufficient quantity to maximize MPS (see [5]), the resulting hyperaminoacidemia/hyperinsulinemia is sufficient to not only maximize MPS, but also fully inhibit MPB. These findings corroborate earlier work by Greenhaff and colleagues (2008) who demonstrated that low concentrations (5 mU/L) of insulin are *required* to mediate a maximal amino acid induced stimulation of leg protein synthesis, and that increasing plasma insulin up to 30 mU/L was required to reduce leg protein breakdown by over 50% and increase net protein balance, but concentrations above this were not further inhibitory for protein breakdown [38]. It is important to note that although CHO may not be fundamentally important in altering net protein balance after resistance exercise when adequate protein is provided, muscle glycogen is reduced following resistance exercise [87,88] and CHO has an important role in muscle glycogen resynthesis and is therefore useful to enhance recovery from training [89].

## **Conclusions**

Nutritional interventions designed to maximally stimulate MPS may be useful for those individuals concerned with enhancing skeletal muscle protein accretion, particularly when they are combined with a program of chronic resistance exercise. Factors including protein/EAA dose, protein source, timing of protein ingestion, and amino acid composition appear to impact the magnitude, and possibly duration, of postprandial MPS. Therefore, in terms of current recommendations it appears that consumption of ~ 20–25 g (corresponding to ~ 8–10 g EAA) [5] of a rapidly absorbed protein [6,8,53] may serve to maximally stimulate MPS after resistance exercise in young healthy individuals. Ideal candidates to fulfill such criteria appear to be whey [6,8] or bovine milk [7]. Whether these recommendations hold for individuals outside of ~80-90 kg is unknown and future research is warranted to address this question.

For the elderly, consumption of high quality leucine-rich proteins, such as whey, may be of primary importance to maximize MPS [70,71] although addition of free leucine to meals does not appear to be an effective strategy to enhance muscle mass or strength, at least when measured over 12 weeks [77]. We propose that there is, at least in young individuals, an extended ‘window of anabolic opportunity’ beyond the immediate post-exercise period that persists for at least 24 h, which may result from muscle fibre recruitment dependent enhanced sensitivity of skeletal muscle to amino acid provision. Although amino acids appear to be the primary nutrient effectors of MPS and can independently enhance muscle protein accrual, their effect on MPS, and ultimately muscle growth will be enhanced by chronic resistance exercise.

## Abbreviations

CHO, Carbohydrate; EAA, Essential amino acid; MPB, Muscle protein breakdown; MPS, Muscle protein synthesis.

## **Competing interests**

The authors declare that they have no competing interests.

## **Authors’ contributions**

TACV, NAB, and SMP wrote and edited the manuscript. All authors read and approved the final manuscript.
